# Endogenous Knowledge and Practices in Child Feeding and Nutrition in West Africa: A Review of the Field, Methods, and Perspectives

**DOI:** 10.7759/cureus.116720

**Published:** 2026-09-22

**Authors:** Chérif Issifou, Waliou Bodounrin Ayanda Amoussa Hounkpatin, Kurvich Edwin Féliciano Gnele, Irene Medeme Mitchodigni, Brigitte Kaufmann

**Affiliations:** 1 Faculty of Agronomic Sciences, University of Abomey-Calavi, Abomey-Calavi, BEN; 2 Healthy Diets, World Vegetable Center, West and Central Africa (WCA), Cotonou, BEN; 3 Transdisciplinary and Social-ecological Landuse Research, German Institute of Tropical and Subtropical Agriculture, Witzenhausen, DEU

**Keywords:** child feeding, endogenous knowledge and practices, herbal tea, malnutrition, nutrition, west africa

## Abstract

Interventions against malnutrition in West Africa integrating endogenous knowledge and practices are recognized as innovative and effective solutions. Given the persistence of child malnutrition, documenting this knowledge and practices would provide a strong basis for developing and implementing interventions. This systematic review aims to provide an overview of endogenous knowledge and practices in child feeding and nutrition among mothers in West Africa. Articles published between January 2014 and February 2025 were collected in AGORA, Scopus, PubMed, Google Scholar, and Web of Science databases. The selection was based on many criteria, such as "articles focusing on endogenous knowledge and practices". Studies' risk of bias was assessed using the Joanna Briggs Institute tools, and the data were synthesized through thematic content analysis. Out of 680 collected, six articles were retained and were essentially cross-sectional ethnographic studies. Five topics were addressed for both knowledge and practices: breastfeeding, complementary feeding, health, hygiene, and care. Breastfeeding practices were characterized by the intake of galactagogue foods or herbal teas. For complementary feeding practices, the age of food introduction was documented. In addition, the age and methods of breastfeeding cessation were described. Regarding health, mothers' knowledge of malnutrition and associated diseases as well as the herbal teas used for treatment was reported. In terms of hygiene and child care practices, drinking water treatment and skin care of newborns were documented. These results demonstrate the existence of a large heritage in terms of endogenous knowledge and practices. In order to guide the design of more appropriate projects, research should advance in evaluating the effectiveness of existing practices, particularly those concerning herbal teas.

## Introduction and background

Endogenous knowledge and practices refer to knowledge and practices experienced by society as an integral part of its heritage, as opposed to exogenous knowledge, which is still perceived, at least at this stage, as part of another value system [1]. They are a body of intrinsic knowledge and practices rooted in the historical and biocultural fiber of peoples [2]. These also involve traditional knowledge and practices, which, like endogenous ones, refer to tradition as a heritage of a society [3]. According to Akaffou [2], endogenous knowledge and practices provide an adequate basis for the development of innovative alternatives or solutions adapted to the local context in order to adequately respond to a concern.

Child malnutrition remains a major concern across Africa, particularly in West Africa, where it is recognized as a public health problem in several countries [4]. In Africa, 30.3% of children under five years of age are stunted, and 5.4% are wasted in 2025. Particularly in West Africa, the situation is equally worrying, with 29.7% of children under five years of age stunted and 6.5% wasted. This prevalence of wasting is highest in Africa, reflecting the severity of malnutrition in this region. In terms of micronutrient deficiency, the situation is severe among women and children. In West Africa, more than two out of three children suffered from anemia in 2023 [5,6]. This situation has a negative impact on West African countries, limiting their development.

However, a wide range of endogenous resources are available that could contribute to finding a sustainable solution to the problem of child malnutrition [7-9]. These solutions, based on local food resources and biodiversity, improve nutrition while strongly influencing the transition to sustainable and resilient food systems [10]. Furthermore, Dewey and Adu-Afarwuah [11], in their review of complementary feeding interventions in developing countries, demonstrated that integrating endogenous resources considerably enhances the effectiveness of such interventions. An Ethiopian study conducted by Gizaw et al. [12] on the effectiveness of nutrition intervention promoting traditional nutritional solutions showed an improvement in mothers' nutritional knowledge, attitudes, and self-efficacy in combating malnutrition. In addition, Aubel et al. [13] demonstrated in their studies conducted in Senegal that the use of traditional knowledge in nutrition projects, through grandmothers, positively influences mothers' practices for the well-being of their children.

These are the indicators that endogenous knowledge and practices represent an excellent alternative solution to solve the child malnutrition problem. But most of the development projects and programs underestimate the impact of endogenous knowledge in improving child health [9]. Given their importance, documenting endogenous knowledge and practices is a crucial step in developing viable and impactful projects and programs in child feeding and nutrition.

Despite the existence of previous studies on endogenous knowledge and practices, these often focus on limited aspects of child feeding and nutrition. To our knowledge, no current synthesis comprehensively identifies and summarizes this knowledge and practices across the various areas of child feeding and nutrition in West Africa. Consequently, this literature review aims to provide an overview of endogenous knowledge and practices of mothers in terms of child under-five feeding and nutrition in West Africa.

## Review

Methods

The systematic review was conducted to synthesize the existing literature on endogenous knowledge and practices of mothers in terms of child feeding and nutrition in West Africa. To achieve the objective of the study, the first three authors carried out research on specific data and used the CADIMA platform (Collaboration for Environmental Evidence (CEE) and Julius Kühn-Institut (JKI), Quedlinburg, Saxony-Anhalt, Germany) according to the following methodology.

Eligibility Criteria for Article Selection and Source of Information

The inclusion criteria used in this study were (1) studies conducted in West Africa or including West African countries, (2) articles addressing child feeding and nutrition, (3) articles addressing mothers' knowledge or practices, and (4) articles focusing on endogenous, traditional, or positive deviant knowledge and practices. Articles with the following characteristics were excluded: (1) publication date prior to 2014 and (2) articles that were completely inaccessible. The search was conducted in February 2025 in the following databases: AGORA, Scopus, PubMed, Google Scholar, and Web of Science.

Search

The search syntax used was formulated using the Population, Interventions, Comparison, and Outcomes (PICO) method. Considering the scope of our research, only the population and intervention (PI) components were applied. Thus, the following syntaxes have been used for Google Scholar, Web of Science, and Scopus: (caregiver* OR mother* OR grandmother* OR women* OR guardian* OR matron*) AND ("indigenous knowledge" OR "local knowledge" OR "traditional knowledge" OR "cultural knowledge" OR "intrinsic knowledge" OR "community-based knowledge" OR "heritage knowledge" OR "autochthonous knowledge" OR "Native knowledge" OR "ancient knowledge" OR "innovative knowledge" OR "unusual knowledge" OR "uncommon knowledge" OR "positive deviant knowledge" OR "indigenous practice" OR "local practice OR "traditional practice" OR "cultural practice" OR "intrinsic practice" OR "community-based practice" OR "heritage practice" OR "autochthonous practice" OR "native practice" OR "ancient practice" OR "innovative practice" OR "unusual practice" OR "uncommon practice" OR "positive deviant practice" ) AND ( "child nutrition" OR "infant nutrition" OR "toddler nutrition" OR "kid nutrition" OR "baby nutrition" OR "childhood nutrition" OR "child feeding" OR "infant feeding" OR "toddler feeding" OR "kid feeding" OR "baby feeding" OR "childhood feeding" OR breastfeeding OR "complementary feeding" OR "nutritional status" OR "nutritional value" OR water OR sanitation OR hygiene OR weaning OR "child care" OR "infant care" OR "toddler care" OR "kid care" OR "baby care" OR "childhood care" OR "health care" OR "young child development") AND (Benin* OR "Burkina-faso" OR "Cape-verde" OR "Ivory Coast" OR Gambia* OR Ghana* OR Guinea* OR "Guinea Bissau" OR Liberia* OR Mali* OR Mauritania* OR Niger* OR Nigeria* OR Senegal* OR "Sierra leone" OR Togo* OR "western Africa"). The syntax used in AGORA was shorter due to the limitation on the number of search terms in the database. For PubMed, certain terms were removed because they did not correspond to Medical Subject Headings (MeSH) themes (Appendix 1).

Selection of Articles

All search results were exported in CADIMA, except for those from Google Scholar, where exportation was limited to the results from the first 10 pages, since beyond that the results were no longer relevant. Then, the selection of documents was carried out by authors 1 and 3 in four stages. Firstly, duplicates were removed. Secondly, the titles and abstracts of all documents were screened according to the inclusion and exclusion criteria. Thirdly, the full texts of the selected studies were reviewed. Finally, the quality of the studies was assessed through Joanna Briggs Institute tools. Both English and French papers were included (Figure 1).

**Figure 1:**
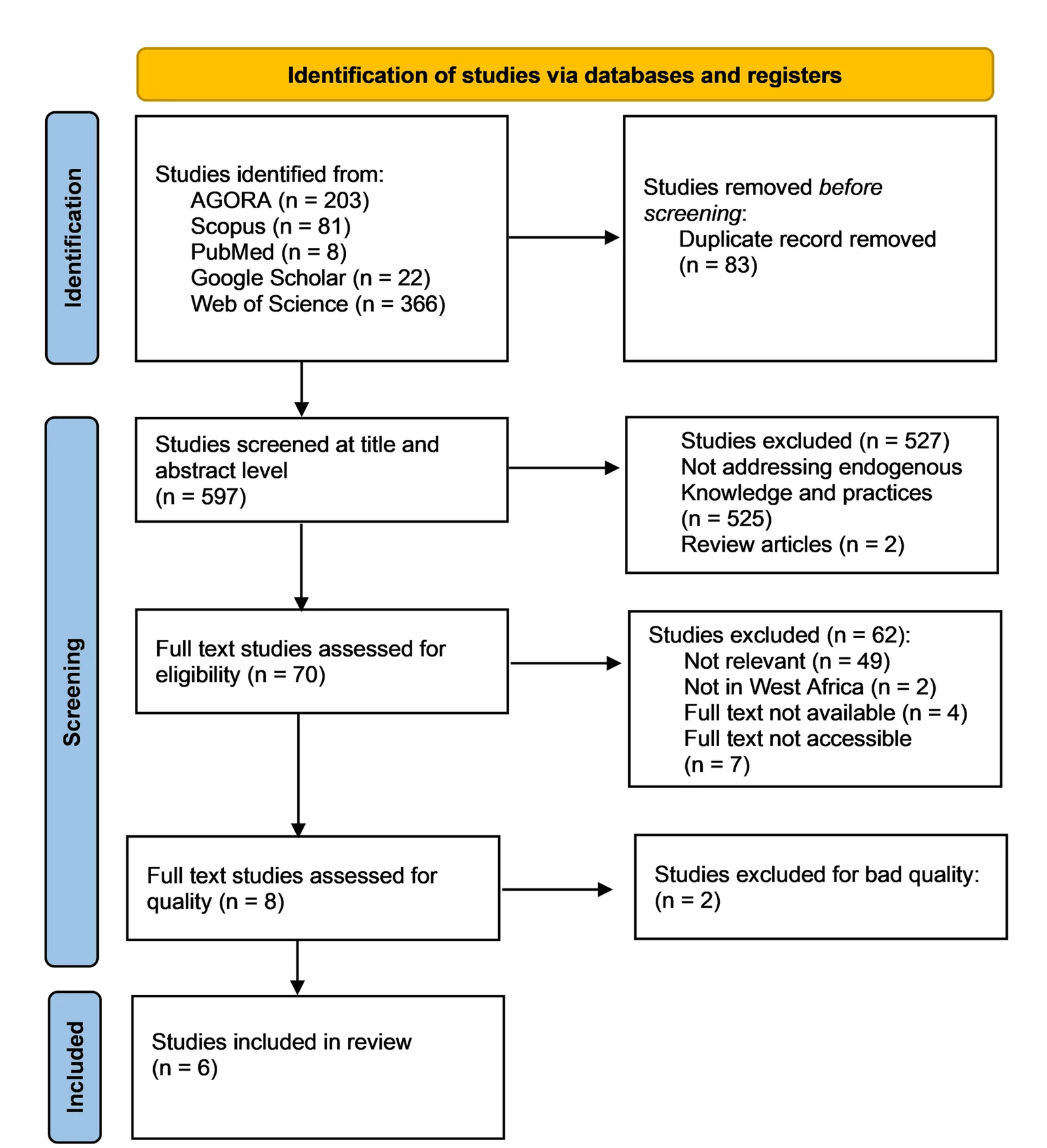
Search flow diagram of publications through the different phases of the review

Data Collection Process and Data Items

Data extraction was carried out by authors 1 and 3 according to an extraction sheet. This included the following aspects: (1) the title of the study, (2) the authors, (3) the year of publication, (4) the country and region of study, (5) the area of child feeding and nutrition covered by the study, (6) the methodology of the study, (7) the results of the study, and (8) the recommendations and perspectives. The discrepancies have been discussed with a second author.

Synthesis Methods and Risk of Bias in Individual Studies

The data were analyzed using thematic content analysis to identify the themes addressed in the selected studies. Text from the "Results" sections of the primary studies was coded inductively. Related codes were grouped to construct descriptive themes reflecting the primary authors' original findings. Descriptive themes were interpreted to generate overarching analytical insights. A summary by theme was prepared in both text and table formats. The quality of the studies was assessed using Joanna Briggs Institute tools. Two independent researchers evaluated the quality of the selected articles. Titles, abstracts, and full texts were independently assessed. Any disagreements were resolved through discussion and consensus. Articles scoring over 50% were considered to be of good quality, while those that received a lower score were excluded (Appendix 2). This cutoff threshold ensured that studies with critical methodological deficiencies were excluded from the final synthesis [14].

Results

Overview of Search

A total of 680 documents were collected from the various databases, and 597 were retained after the elimination of duplicates. After title and abstract screening, 70 documents were selected, and eight studies were retained during full-text screening. Two studies were excluded for low quality. Finally, six articles were selected at the end of the process.

Study Setting and Design

Table 1 presents the characteristics of the selected articles. The studies were conducted in the following West African countries: Benin, Ghana, the Republic of Niger, and Nigeria. Two articles were cross-sectional, and four were ethnographic qualitative studies. The studies were published between 2015 and 2024. The following topics are covered: breastfeeding, complementary feeding, hygiene, health, and care. Five studies addressed mothers' practices, and one addressed both mothers' knowledge and practices.

**Table 1:** Summary of the selected studies *IDs are the serial numbers of articles assigned by the CADIMA platform prior to the selection process. ^1^This study also covers other African countries, including Ethiopia and Tanzania.

ID*	Author	Country	Study type	Areas of feeding and nutrition addressed	Knowledge or practices
31	Tahiru et al. [15]	Ghana	Qualitative and ethnographic	Breastfeeding, complementary feeding, care	Practice
38	Vissoh et al. [16]	Benin	Quantitative cross-sectional	Health	Knowledge and practice
124	Garba et al. [17]	Republic of Niger	Quantitative cross-sectional	Complementary feeding, health	Practice
134	Amare et al. [18]^1^	Nigeria	Qualitative and ethnographic	Care	Practice
257	Oloko and Ekpo [19]	Nigeria	Qualitative and ethnographic	Complementary feeding, breastfeeding, hygiene	Practice
596	Lawal et al. [20]	Nigeria	Qualitative and ethnographic	Breastfeeding	Practice

Knowledge

The endogenous knowledge reported by the studies primarily concerns the management of diarrhea risks when weaning and their causes. Secondly, they address the causes and consequences of child malnutrition and associated diseases.

Regarding diarrhea, studies reported that mothers were aware of the risks during the cessation of breastfeeding and attributed them mainly to teething [19]. For child malnutrition, poverty, chronic diseases, an unbalanced diet, and insufficient food intake were identified by mothers as the main causes. Signs of malnutrition such as excessive weight loss, bloating, brittle hair, and delayed growth have been mentioned. The consequences of malnutrition reported included the following: delayed growth, weakness in the child's body or immune system, the child takes longer to learn to move and speak, the child experiences difficulties in understanding and learning things, and the child catches diseases like diarrhea and respiratory tract infections, which can have symptoms such as cough, sore throat, fever, runny nose, and difficulty breathing. All mothers consistently reported the use of plants in the traditional management of malnutrition [16].

Practices

Breastfeeding practices: Breastfeeding practices included the frequency of breastfeeding and the use of galactagogues by mothers. Breastfeeding was traditionally done on demand. The need to breastfeed is recognized by mothers when babies cry. In addition, grandmothers emphasized the existence of an intuitive link between mother and child, indicating the need to be breastfed (breasts were full of milk, breast pain). The ability to recognize these intuitive signs is characteristic of a good mother [15].

Breastfeeding mothers use traditional recipes and galactagogue plants to stimulate milk production (Table 2). Tahiru et al. [15] in Ghana mentioned liquid galactagogue foods (tea, soy milk drink, millet drink (zim koom)), semi-liquid (kenkey frozen, mpanpa, peanut soup), and solid (Tuo-Zaafi). In terms of plants, Garba et al. [17] and Lawal et al. [20] have documented in Niger and Nigeria several families (24) of plants. The family of Fabaceae was the most dominant (Table 3).

**Table 2:** Galactagogue recipes in Ghana Source: [15]

Food	Recipes	Description	Ingredients
Tea	Liquid	-	Milo and milk powder
Kanwa koko	Semi-solid	Unfermented millet porridge	Pearl millet + spices (ginger, cloves, pepper)
Mpanpa	Semi-solid	Cereal porridge	Corn, millet, rice, soy + spices
Tuo-Zaafi with peanut soup	Solid	Thick porridge made from corn flour or millet as an accompaniment to peanut soup	Corn or millet flour, peanuts or peanut paste, vegetables (onion, tomatoes, garlic, chili peppers), meat or fish, spices
Ice kenkey	Semi-solid	Kenkey (steamed dumpling made from fermented corn flour)	Kenkey, water, sugar, milk, or peanuts
Zoomkoom	Liquid	Millet-based drink	Pineapple, sugar, ginger, millet flour, tamarind
Soy milk	Liquid	Soy-based drink	Soybean, water, sugar

**Table 3:** Galactogenic plants and parts used Sources: [17,20]

Plants species	Family	Part of the plant used	How to use
*Vernonia amygdalina* Delile	Asteraceae	Bark	Maceration
*Tamarindus indica* (L.)	Fabaceae	Pod	Maceration
*Hibiscus asper* Hook.f	Malvaceae	Leaves	Cooking
*Vigna unguiculata* (L.) Walp	Fabaceae	Shrubs	Powder
*Cleome gynandra* L.	Cleomaceae	Whole plant	Cooking
*Balanites aegyptiaca* (L.) Delile	Balanitaceae	Leaves	Maceration
*Ipomoea batatas* (L.) Lam	Malvaceae	Leaves	Cooking
*Crotalaria retusa* L.	Apocynaceae	Whole plant	Decoction
*Commelina forskaolii* Vahl	Commelinaceae	Leaves	Powder
*Maerua angolensis* DC.	Capparaceae	Leaves	Decoction
*Solanum incanum* L.	Solanaceae	Leaves	Maceration
*Acacia nilotica* (L.) Delile	Fabaceae	Leaves	Maceration
*Phoenix dactylifera* L.	Arecaceae	Fruit	Direct
*Zea mays* L.	Poaceae	Shrubs	Cooking
*Olea europaea* L.	Oleaceae	Leaves	Decoction
*Byrsocarpus coccineus* Schum. and Thonn.	Connaraceae	Whole plant	Powder
*Andropogon gayanus* Kunth	Poaceae	Leaves	Decoction
*Arachis hypogaea* L.	Fabaceae	Leaves	Decoction
*Guiera senegalensis* J.F. Gmel	Combretaceae	Leaves	Powder
*Sesamum indicum* L.	Pedaliaceae	Shrubs	Direct
*Diospyros mespiliformis* Hochst. ex A. DC	Ebenaceae	Bark	Maceration
*Euphorbia hirta* L. Parts	Euphorbiaceae	Whole plant	Powder
*Moringa oleifera* Lam.	Moringaceae	Leaves	Cooking
*Centaurea perrottetii* DC.	Asteraceae	Whole plant	Maceration
*Ricinus communis* L., oleum	Euphorbiaceae	Leaves	Cooking
*Glycine max* (L.) Merr	Fabaceae	Shrubs	Powder
*Tephrosia bracteolata* Guill. & Perr.	Fabaceae	Fruit	Powder
*Cocos nucifera* L.	Arecaceae	Fruit	Direct
*Senna occidentalis* (L.) Link	Fabaceae	Leaves	Decoction
*Carica papaya* L.	Caricaceae	Leaves	Decoction
*Euphorbia lateriflora* Schumach.	Euphorbiaceae	Whole plant	Powder
*Cassia arereh* Delile	Leguminoceae	Rhizome	Maceration
*Lannea microcarpa* Engl. & K. Krause	Anacardiaceae	Bark	Maceration
*Azadirachta indica* A. Juss.	Meliaceae	Leaves	Decoction
*Boscia salicifolia* Oliv.	Capparaceae	Leaves	Powder
*Pennisetum glaucum* (L.) R. Br.	Poaceae	Shrubs	Cooking
*Cyperus esculentus* L. var. sativus Boeck	Cyperaceae	Shrubs	Direct
*Sorghum bicolor* L.	Poaceae	Shrubs	Cooking
*Cassia sieberiana* DC.	Leguminoceae	Pod	Maceration
*Momordica balsamina* L.	Cucurbitaceae	Leaves	Powder

Complementary feeding practices: Regarding complementary feeding practices, the studies focused on the timing of the introduction of solid foods as well as the timing of the cessation of breastfeeding and the foods used during this period of child life. The introduction of food depends on the child's maturity and can occur between the first and second months of the baby's life [15].

Basically, in West African regions, the cessation of breastfeeding occurred more than 18 months after the child's birth. In a study conducted in Nigeria by Oloko and Ekpo [19], traditional foods used for this purpose (Table 4) were based on cereal (millet) and legumes (groundnut and cowpeas). The choice made by mothers is strongly influenced by the family, mainly grandmothers [19].

**Table 4:** Traditional foods used by mothers during the cessation of breastfeeding in Nigeria Source: [19]

Food	Meal	Description
Dawa (*Pennisetum glaucum*), gyero (*Sorghum bicolor*)	Fura da nunu	Millet/sorghum balls and fresh cow milk
	Danbu	Grounded, cooked, and shredded millet/sorghum
	Tuwon dawa/gyero	Thick paste of grounded millet/sorghum. Eaten with soup
	Kamun dawa/koko	Millet/sorghum pap. Sometimes local spices are added
	Kunu	Gruel made up of millet/sorghum and sprouted rice (sometimes dried sweet potatoes)
Shinkafan gida (*Oryza glaberrima*)	Tuwon shinkafa	Thick paste of local rice. Eaten with soup
	Shinkafa	Boiled rice eaten with soup
Wake (*Vigna unguiculata*)	Gwaten wake	Beans porridge, cooked with local condiments
	Alele	Peeled grounded boiled beans usually grounded with bell pepper and onions; sometimes, crayfish/fish/liver/egg is added
	Kosai	Peeled and grounded beans. Usually fried
	Danwake	Ground bean balls (mixed with maize flour as a thickener). Usually eaten with groundnut oil and spices if desired
Gyeda (*Arachis hypogaea*)	Kunun gyeda	Pap made up of ground nut and local spices. Usually thickened with rice or millet paste

Health*: *In terms of health, herbal tea recipes used to cure children's illnesses were mentioned in selected articles. Traditional herbal tea recipes were used to treat malnutrition and associated diseases like malaria, cough, diarrhea, etc. (Appendix 3). All herbal tea recipes were 174 and were composed of 1-5 plants. The most frequent recipes used were the monoplant recipes. The Fabaceae family was the most dominant among 40 families of plants used by mothers. The plant organs prepared for the treatment and the preparation method were mostly the leaves and infusion, respectively [16,17].

Hygiene: Only water hygiene was reported. Oloko and Ekpo [19] revealed that mothers in Nigeria used two techniques to ensure the purity of the drinking water. Rainwater was collected, and when it was unavailable, mothers treated water by heating it before use.

Child care:* *In terms of child care, the studies analyzed report on body care. Amare et al. [18] described the emollients used by mothers in Nigeria, the reason for their use, and the timing of their use. Emollients were used by mothers in Nigeria mainly to make the skin soft, smooth, attractive, healthy, strong, and rash-free. In addition, they can keep the baby warm, soften/strengthen the joints/bones, shape the baby, ensure flexibility, promote growth, facilitate weight gain, and improve the baby's sleep. These emollients were divided into four categories, including vegetable oils/baby oils, lotions, medicinal creams, and balms. Vegetable oils include peanut oil, shea butter, olive oil, and mahogany or neem oil; baby lotions; creams (herbal creams); and balms (mentholatum). They were applied by the experienced women of the family after the first bath, immediately in the first days of birth. Then, young mothers were allowed to continue the application of emollients after bathing their babies.

Discussion

The objective of this literature review was to provide an overview of endogenous knowledge and practices of mothers in terms of child feeding and nutrition in West Africa. The results first show mothers' knowledge in terms of health. Secondly, it presented mothers' practices related to breastfeeding, complementary feeding, health, hygiene, and care.

Malnutrition and Associated Disease Knowledge

The studies showed that endogenous knowledge was related to the signs, causes, and consequences of child malnutrition and associated pathologies, as well as the prevention of diarrhea.

For mothers, poverty, insufficient food intake, chronic diseases, and unbalanced diets were the main causes of malnutrition. These results are fully in line with the conceptual framework of malnutrition described by the United Nations Children's Fund (UNICEF) [21]. In addition, poverty is a key determinant of malnutrition, as it limits the coverage of children's basic nutritional needs [22].

Signs of malnutrition such as excessive weight loss, bloating, brittle hair, and delayed growth reported by mothers were consistent with marked muscle atrophy, abdominal distension, dense dry hair, and hypopigmented hair characteristic of severe wasting [23]. In addition, diarrhea is associated with a small decrease in growth and weight loss and therefore has an impact on child health [24,25]. According to the mothers, the consequences were physical and immune weaknesses, delayed motor and mental development, and diseases such as diarrhea and respiratory infections. These factors are similar to those listed by Adeyeye et al. [26] as effects of malnutrition.

According to mothers, the risk of diarrhea during the cessation of breastfeeding is commonly due to teething. This perception is consistent with those reported in a study conducted in Africa on the perception of dental symptoms [27]. However, DenBesten [28] reports that this association is not always clear and that such beliefs should be discouraged to ensure that mothers take diarrhea more seriously during teething.

Breastfeeding Practice and Galactagogue Recipes

Breastfeeding on demand is the main practice used by mothers to feed their babies. During this practice, the mother-child couple's emotional bond is very important in the recognition of the child's needs. Breastfeeding on demand is highly recommended by the World Health Organization (WHO) as a good practice for children under two years of age [29]. It also ensures that the child's needs are covered, reduces the risk of childhood illness, contributes to the baby's psychomotor and cognitive development, and reduces the risk of breast cancer, diabetes, hypertension, and cardiovascular diseases for the mother [30].

In order to have sufficient breast milk, mothers used several traditional methods to increase it. These methods involve using galactagogue recipes and plants mainly from the Fabaceae family to stimulate breast milk production. The galactagogue recipes listed were mainly composed of cereals and legumes known for their high energy and protein content [31]. Energy and protein contribute strongly to breast milk production [32]. Since breast milk production consumes significant amounts of energy, these recipes are suitable sources for providing the energy needed for this process [32]. Legumes contribute to breast milk production through their proteins and phytoestrogens, which have hormonal effects [32,33].

The galactogenic properties of plants from the Fabaceae family have been proven by several authors [20]. They contain chemical compounds such as alkaloids, flavonoids, and saponins that act as stimulators of breast milk production [34].

Food Consumption During Complementary Feeding

Regarding the introduction of complementary foods, studies have shown that this varies depending on the maturity of the child. It can start in the first and second months after birth. Mothers explain this practice by the need for children to consume foods other than breast milk [15]. However, breast milk during the first six months contains all the necessary nutrients and essential immunological elements to cover a child's nutritional needs and protect him against possible diseases. For this reason, WHO recommends exclusive breastfeeding for up to six months. Cost-effective for the household, when practiced correctly, exclusive breastfeeding promotes the harmonious growth of the child [29]. It is therefore important to recommend the promotion of exclusive breastfeeding in the community, particularly its effective practice. Nutritional education targeting mothers on this practice is necessary to promote change in their behavior.

Prior to the six months of exclusive breastfeeding recommended by the WHO, mothers are confronted with childhood illnesses that they are compelled to manage. They use herbal teas, in this context, to overcome these illnesses. However, the uncontrolled use of these herbal teas could harm the children rather than cure them. The main challenge is the inadequate hygiene practices involved in preparing and consuming these herbal teas. Darkwah et al. [35], in their work on microbial contamination of herbal teas prepared in Ghana, reported a high risk of contamination by pathogens such as *Escherichia coli *and *Enterobacter *spp. Moreover, the doses of these herbal teas administered by mothers to children can, in some cases, be dangerous [36].

In general, mothers start the cessation of breastfeeding at 18 months after childbirth, which is consistent with the WHO recommendation [29]. The choice regarding foods for complementary feeding was strongly influenced by the family, especially grandmothers and relatives. A study conducted in Brazil highlighted that grandmothers were key references in feeding children [37].

Drinking Water Treatment

Concerning hygiene, the focus was on drinking water. In the rural setting of Nigeria, some mothers use heating and collection of rainwater to ensure the safety and purity of the drinking water. The heat treatment of water has been documented as an effective traditional method of water purification. Indeed, the heat treatment applied during this method eliminates the majority of pathogens [38]. For rainwater, studies have shown that it can be a good source of drinking water for households [39]. However, this water may contain particles, bacteria, and air pollutants that can cause diseases. WHO recommends filtering out or treating rainwater before using it as drinking water in households [40]. Although rainwater is not free of pathogenic microbes, its consumption reduces the risk of diarrheal diseases compared to other unimproved water sources [39].

Treatment of Malnutrition and Associated Diseases

In terms of health, studies have shown that plants from the Fabaceae family were the most dominant for the treatment of malnutrition and associated diseases such as malaria, diarrhea, and lack of appetite. The Fabaceae family was recognized as a group of plants used in the treatment of malaria [41]. In their respective studies on the traditional knowledge of antimalarial plants and on the antiplasmodial compounds of plants, Dénou et al. [42] proved the antimalarial properties of *Indigofera leptoclada* Harms and *Gardenia sokotensis* Hutch.

*Senna occidentalis* (L.) Link, of the Fabaceae family, has been documented as an anti-diarrheal drug in the review of Singh et al. [43]. Its composition of tannins and saponosides provides anti-inflammatory, antimicrobial, and astringent properties to fight diarrhea.

While diarrhea causes the loss of nutrients after digestion, lack of appetite occurs upstream by reducing the amount of food ingested by children. Among the herbal teas revealed in this study, *Cassia sieberiana *DC. possesses bioactive compounds that may help alleviate lack of appetite [44]. Archer et al. [45] have shown that this plant contains alkaloids and flavonoids that are relevant in stimulating appetite.

In addition to addressing diseases associated with malnutrition, Fabaceae family plants were also used to directly combat malnutrition [46]. This family includes several mentioned plants that are rich in essential nutrients for the body. Plants such as *Glycine max* (soybeans) and *Arachis hypogaea* are full of energy, proteins, and micronutrients, accessible to poor people [31]. The seeds of *Arachis hypogaea *and *Glycine max* contain 20.4-25.2 g/100 g and 12.9-35.9 g/100 g protein, 531-585 kcal/100 g and 379-395 kcal/100 g energy, 2.3-8.2 mg/100 g and 3.9-8.3 mg/100 g in iron, and 2.61-3.65 mg/100 g and 4.73-4.87 mg/100 g in zinc, respectively. These plants represent a valuable nutrient source for improving the nutritional status of children.

The articles reviewed reported on the use of emollients by mothers for infant skin care. These emollients can be used in the treatment of child malnutrition. In the treatment of severe acute malnutrition, the application of a topical emollient on the skin of children has a beneficial effect [47]. Indeed, these emollients preserved the integrity of the skin and thus prevented infections in newborns that could impact their nutritional status [48].

Perspectives

Most of the studies reviewed were cross-sectional ethnographic in design. However, none employed a participatory approach, which is recognized for enhancing target population engagement and providing deeper insights into endogenous practices [49]. Using this approach in future research could facilitate the discovery of more endogenous knowledge and practice relevant to improving child nutrition. Although certain aspects of infant nutrition have been explored in these studies, other aspects could certainly be addressed to enrich endogenous knowledge and practices relating to child feeding and nutrition. Those of hygiene and care need to be further investigated to document endogenous knowledge and practices on food and personal hygiene, as well as parent-child relationships that promote harmonious child development. In terms of breastfeeding and health, several herbal teas have been documented as galactagogues and for combating malnutrition and associated diseases. Future studies should go beyond this by investigating their effectiveness. In addition, the knowledge and practices documented are mainly focused on the treatment and not on the screening of malnutrition. Thus, certain signs and symptoms have been mentioned for the detection of malnutrition. However, there is a need to establish a local and effective screening system to enable the early identification of child malnutrition. Considering the inadequacies of health systems in developing countries coupled with limited resources, the investigation of local methods of malnutrition management became imperative [50].

## Conclusions

The objective of this review was to provide an overview of endogenous knowledge and practices in child feeding and nutrition among mothers in West Africa. Out of 680 collected, six articles were finally retained and addressed the following topics: breastfeeding, complementary feeding, health, hygiene, and care. Breastfeeding on demand was practiced in the studies analyzed. This practice is supported by the intake of galactagogue foods or herbal teas. In terms of complementary feeding, the introduction of food started before the second month of birth with liquid foods. The cessation of breastfeeding occurred after 18 months with specific foods based on cereals and legumes. Fabaceae family plants were mainly used for herbal teas to treat malnutrition and associated diseases. In terms of hygiene and child care, the use of heat treatment to purify water and the use of emollients for the protection of the skin of newborns were documented. These findings highlight the existence of endogenous knowledge and practices that can be leveraged to enhance child nutrition and address malnutrition. But they also emphasize the need to continue documenting more endogenous knowledge and practices in the areas covered by this study, particularly in care and hygiene. However, certain practices, such as the early introduction of solid foods, should be avoided. Furthermore, the use of the aforementioned herbal teas must be approached with caution, given their potential toxicity depending on the dose and proper hygiene practices. In addition, these findings are derived from a limited evidence base consisting of six studies across four West African countries. Consequently, caution must be exercised, and these results cannot be broadly generalized to all of West Africa, as local practices, healthcare access, and traditional frameworks vary significantly across the region. Furthermore, it is crucial to advance research by evaluating the effectiveness of practices (in particular herbal teas used as galactagogues and in the treatment of malnutrition and associated diseases) and by co-developing with the community's new knowledge and practices. This approach could be critical for improving early detection of malnutrition in order to provide rural populations with tools to address child malnutrition.

## Appendices

Appendix 1

Syntax Used for Search Strategy

PubMed: ("Caregiver" OR "Mother" OR "Grandmother" OR "Women" OR "Guardian" OR "Matron") AND ("Indigenous Knowledge" OR "Local Knowledge" OR "Traditional Knowledge" OR "Cultural Knowledge" OR "Intrinsic Knowledge" OR "Community-based Knowledge" OR "Native Knowledge" OR "Ancient Knowledge" OR "Innovative Knowledge" OR "Unusual Knowledge" OR "Uncommon Knowledge" OR "Indigenous Practice" OR "Local Practice" OR "Traditional Practice" OR "Cultural Practice" OR "Community-based Practice" OR "Heritage Practice" OR "Ancient Practice" OR "Innovative Practice" OR "Unusual Practice" OR "Uncommon Practice" OR "Positive Deviant Practice") AND ("Child Nutrition" OR "Infant Nutrition" OR "Toddler Nutrition" OR "Childhood Nutrition" OR "Nutritional Status" OR "Nutritional Value" OR "Water" OR "Sanitation" OR "Hygiene" OR "Weaning" OR "Breastfeeding" OR "Complementary Feeding" OR "Child Care" OR "Infant Care" OR "Toddler Care" OR "Young Child Development") AND ("Benin" OR "Burkina Faso" OR "Cape Verde" OR "Ivory Coast" OR "Gambia" OR "Ghana" OR "Guinea" OR "Guinea Bissau" OR "Liberia" OR "Mali" OR "Mauritania" OR "Niger" OR "Nigeria" OR "Senegal" OR "Sierra Leone" OR "Togo" OR "Western Africa")

AGORA: « (Caregiver* OR mother* OR women*) AND (Endogenous* OR local* OR traditional*) AND Practice* AND Knowledge* AND (Child* OR infant* OR kid* OR baby* OR Toddler) AND (Nutrition* OR Feeding* OR Breastfeeding* OR “Complementary feeding" OR Hygiene* OR care) AND ("Benin" OR "Burkina Faso" OR "Cape Verde" OR "Ivory Coast" OR "Gambia" OR "Ghana" OR "Guinea" OR "Guinea Bissau" OR "Liberia" OR "Mali" OR "Mauritania" OR "Niger" OR "Nigeria" OR "Senegal" OR "Sierra Leone" OR "Togo") » AGORA's syntax has been reduced compared to other databases due to the limited number of words that AGORA's search bar can accept.

Appendix 2

Quality Assessment of the Selected Articles

**Table 5:** Quality assessment of the selected articles PCA: principal component analysis

Evaluator 1: Ricardo Ayi (MSc)																	
Record number	Study type	Date	Author	Year	Quality criteria										Percentage of percentage of compliance with quality criteria (number of Yes/(Yes + No)	Overall appraisal	Comments (including reason for exclusion)
					Is there congruity between the stated philosophical perspective and the research methodology?	Is there congruity between the research methodology and the research question or objectives?	Is there congruity between the research methodology and the methods used to collect data?	Is there congruity between the research methodology and the representation and analysis of data?	Is there congruity between the research methodology and the interpretation of results?	Is there a statement locating the researcher culturally or theoretically?	Is the influence of the researcher on the research, and vice versa, addressed?	Are participants, and their voices, adequately represented?	Is the research ethical according to current criteria or for recent studies, and is there evidence of ethical approval by an appropriate body?	Do the conclusions drawn in the research report flow from the analysis, or interpretation, of the data?			
31	Qualitative study	08/06/2025	Rafatu Tahiru, Mary Amoako, and Charles Apprey	2024	Yes	Yes	Yes	Yes	Yes	No	No	Yes	Yes	Yes	80	Include	The article is generally of good quality, but it lacks aspects of reflexivity that describe the researcher's influence on the data collected
134	Qualitative study	08/06/2025	Yared Amare, Donat D. Shamba, Fatuma Manzi, Margaret H. Bee, Babatunji A. Omotara, Ruth B. Iganus, Ebunoluwa A. Adejuyigbe, Adetanwa L. Odebiyi, Jolene Skordis-Worrall, and Zelee E. Hill	2015	Yes	Yes	Yes	Yes	Yes	No	No	Yes	Yes	Yes	80	Include	The article is generally of good quality, but it lacks aspects of reflexivity that describe the researcher's influence on the data collected
275	Qualitative study	08/06/2025	Majing Oloko and Regina Ekpo	2018	Yes	Yes	Yes	Yes	Yes	No	No	Yes	No	Yes	70	Include	The article is generally of good quality, but it lacks aspects of reflexivity that describe the researcher's influence on the data collected. Also, there is no ethical approval
596	Qualitative study	08/06/2025	Lawal U, Mohammed RT, Gidado SM, and Kankara SS	2023	Yes	Yes	Yes	Yes	Yes	Yes	Yes	Yes	Yes	Yes	100	Include	Good quality
					Were the criteria for inclusion in the sample clearly defined?	Were the study subjects and the setting described in detail?	Was the exposure measured in a valid and reliable way?	Were objective, standard criteria used for measurement of the condition?	Were confounding factors identified?	Were strategies to deal with confounding factors stated?	Were the outcomes measured in a valid and reliable way?	Was appropriate statistical analysis used?					
38	Cross-sectional study	09/06/2025	Ahamidé Carel Sosthène Vissoh, Jean Robert Klotoé, Lauris Fah, Eric Agbodjento, Hornel Koudokpon, Eskyl Togbe, Souad Saïdou, and Victorien Dougnon	2024	Yes	Yes	Yes	Yes	Not applicable	Not applicable	Yes	Yes	-	-	100	Include	Good quality
124	Cross-sectional study	09/06/2025	Rahila Hama Garba, Lewamy Mamadou, Yaou Chaibou, Sabo Haoua Seyni, Moussa Idrissa, and Sadou Hassimi	2022	Yes	Yes	Yes	No	Not applicable	Not applicable	Yes	Yes	-	-	83,333333	Include	Good quality
680	Cross-sectional study	09/06/2025	Sandrine E. Kouton, Waliou Amoussa Hounkpatin, Vénérande Y. Ballogou, Jaurès H. Lokonon, and Mohamed M. Soumanou	2017	Yes	No	No	No	Not applicable	Not applicable	Yes	No	-	-	33,333333	Exclude	Insufficient description of the study, subjects, and context and inappropriate statistical analysis
250	Cross-sectional study	09/06/2025	Odefadehan OO, Abiodun OO, Odefadehan OO, and Daniel OO	2020	Yes	No	No	No	Not applicable	Not applicable	Yes	No	-	-	33,333333	Exclude	Insufficient description of the study, subjects, and context, and statistical analysis is not described. The description of methodology is poor
Evaluator 2: Fadilath Chabi Yoruba (MSc)																	
Record number	Study type	Date	Author	Year	Quality criteria										Percentage of percentage of compliance with quality criteria (number of Yes/(Yes + No)	Overall appraisal	Comments (including reason for exclusion)
					Is there congruity between the stated philosophical perspective and the research methodology?	Is there congruity between the research methodology and the research question or objectives?	Is there congruity between the research methodology and the methods used to collect data?	Is there congruity between the research methodology and the representation and analysis of data?	Is there congruity between the research methodology and the interpretation of results?	Is there a statement locating the researcher culturally or theoretically?	Is the influence of the researcher on the research, and vice versa, addressed?	Are participants, and their voices, adequately represented?	Is the research ethical according to current criteria or for recent studies, and is there evidence of ethical approval by an appropriate body?	Do the conclusions drawn in the research report flow from the analysis, or interpretation, of the data?			
31	Qualitative study	20/06/2025	Rafatu Tahiru, Mary Amoako, and Charles Apprey	2024	Yes	Yes	Yes	Yes	Yes	No	No	Yes	Yes	Yes	80	Include	-
134	Qualitative study	20/06/2025	Yared Amare, Donat D. Shamba, Fatuma Manzi, Margaret H. Bee, Babatunji A. Omotara, Ruth B. Iganus, Ebunoluwa A. Adejuyigbe, Adetanwa L. Odebiyi, Jolene Skordis-Worrall, and Zelee E. Hill	2015	Yes	Yes	Yes	Yes	Yes	No	No	Yes	Yes	Yes	80	Include	-
275	Qualitative study	20/06/2025	Majing Oloko and Regina Ekpo	2018	Yes	Yes	Yes	Yes	Yes	No	No	Yes	No	Yes	70	Include	-
596	Qualitative study	21/06/2025	Lawal U, Mohammed RT, Gidado SM, and Kankara SS	2023	Yes	Yes	Yes	Yes	Yes	Yes	Yes	Yes	Yes	Yes	100	Include	-
					Were the criteria for inclusion in the sample clearly defined?	Were the study subjects and the setting described in detail?	Was the exposure measured in a valid and reliable way?	Were objective, standard criteria used for measurement of the condition?	Were confounding factors identified?	Were strategies to deal with confounding factors stated?	Were the outcomes measured in a valid and reliable way?	Was appropriate statistical analysis used?					
38	Cross-sectional study	21/06/2025	Ahamidé Carel Sosthène Vissoh, Jean Robert Klotoé, Lauris Fah, Eric Agbodjento, Hornel Koudokpon, Eskyl Togbe, Souad Saïdou, and Victorien Dougnon	2024	Yes	Yes	Yes	Yes	Not applicable	Not applicable	Yes	Yes	-	-	100	Include	Good quality
124	Cross-sectional study	21/06/2025	Rahila Hama Garba, Lewamy Mamadou, Yaou Chaibou, Sabo Haoua Seyni, Moussa Idrissa, and Sadou Hassimi	2022	Yes	Yes	Yes	No	Not applicable	Not applicable	Yes	Yes	-	-	83,333333	Include	Good quality
680	Cross-sectional study	21/06/2025	Sandrine E. Kouton, Waliou Amoussa Hounkpatin, Vénérande Y. Ballogou, Jaurès H. Lokonon, and Mohamed M. Soumanou	2017	Yes	No	No	No	Not applicable	Not applicable	Yes	No	-	-	33,333333	Exclude	The study did not adequately describe the subjects and the context. The PCA used for the statistical analysis was inappropriate. Regression would have been a better approach. Furthermore, the variables were not measured in a valid and reliable manner
250	Cross-sectional study	21/06/2025	Odefadehan OO, Abiodun OO, Odefadehan OO, and Daniel OO	2020	Yes	No	No	No	Not applicable	Not applicable	Yes	No	-	-	33,333333	Exclude	Insufficient description of the study, subjects, and context, and statistical analysis is not described

Appendix 3

Traditional Plants for the Treatment of Malnutrition and Associated Pathologies

**Table 6:** Traditional plants for the treatment of malnutrition and associated pathologies Sources: [16,17]

Recipe type	Scientific names of plants	Families	Plant organs	What treatment does the recipe target?	Preparation method
Monoplant recipe	*Moringa oleifera* Lam.	Moringaceae	Leaves. Barks. Stems. Roots. Seeds	Stimulation of appetite. Fever. Supply of essential nutrients. Diarrhea. Convulsion. Strengthening of the immune system. Improvement of digestion. Cough. Others	Infusion. Decoction. Trituration. Powder. Maceration
Monoplant recipe	*Phyllanthus amarus* Schumach. & Thonn.	Phyllanthaceae	Leaves	Supply of essential nutrients. Stimulation of appetite. Improved digestion. Strengthened immune system. Cough. Diarrhea	Infusion
Monoplant recipe	*Senna siamea* (Lam.) H.S.Irwin & Barneby	Fabaceae	Leaves. Barks. Fruits. Roots	Stimulation of appetite. Supply of essential nutrients. Diarrhea. Strengthening of the immune system. Fever. Cough. Convulsion. Intake of essential nutrients. Improvement of digestion	Infusion. Decoction. Powder
Monoplant recipe	*Carica papaya* L.	Caricaceae	Leaves. Roots. Twigs. Flowers. Stems. Barks. Fruits. Seeds	Fever. Supply of essential nutrients. Appetite stimulation. Others	Infusion. Trituration. Maceration. Decoction. Powder
Monoplant recipe	*Ocimum gratissimum* L.	Lamiaceae	Leaves	Supply of essential nutrients. Stimulation of appetite. Strengthening of the immune system. Diarrhea. Fever. Improved digestion. Cough. Convulsion	Infusion. Trituration
Monoplant recipe	*Adansonia digitata* L.	Malvaceae	Barks. Leaves. Fruits. Seeds. Fruits. Flowers. Roots. Stems. Branches	Stimulation of appetite. Supply of essential nutrients. Strengthening of the immune system. Improvement of digestion. Fever. Diarrhea	Infusion. Trituration. Maceration. Powder. Decoction
Monoplant recipe	*Khaya senegalensis* (Desr.) A. Juss.	Meliaceae	Barks. Leaves	Stimulation of appetite. Supply of essential nutrients. Strengthening of the immune system. Diarrhea. Improvement of digestion. Convulsion. Cough. Diarrhea. Fever	Infusion. Trituration. Decoction. Powder
Monoplant recipe	*Gymnanthemum amygdalinum* (Delile) Sch.Bip.	Asteraceae	Leaves. Roots. Seeds	Intake of essential nutrients. Appetite stimulation. Digestion improvement. Diarrhea. Fever. Immune system strengthening. Cough. Convulsion. Supply of essential nutrients	Decoction. Infusion
Monoplant recipe	*Vitellaria paradoxa* C.F. Gaertn.	Sapotaceae	Leaves	Stimulation of appetite. Supply of essential nutrients. Fever. Improved digestion. Diarrhea	Infusion
Monoplant recipe	*Anonychium africanum* (Guill. & Perr.) C.E.Hughes & G.P.Lewis	Fabaceae	Leaves	Supply of essential nutrients. Fever. Diarrhea. Appetite stimulation. Digestion improvement. Cough. Convulsion	Infusion
Monoplant recipe	*Momordica charantia* L.	Cucurbitaceae	Leaves. Barks. Roots	Essential nutrients. Appetite stimulation. Fever. Cough. Diarrhea. Immune system boost. Digestion enhancement	Infusion. Maceration
Monoplant recipe	*Mangifera indica* L.	Anacardiaceae	Leaves	Immune system boost. Fever. Diarrhea. Essential nutrients. Diarrhea. Convulsion. Appetite stimulation. Cough. Digestion enhancement	Trituration
Monoplant recipe	*Citrus × aurantiifolia* (Christm.) Swingle	Rutaceae	Leaves. Barks	Supply of essential nutrients. Stimulation of appetite. Improved digestion. Diarrhea. Fever. Convulsion. Cough. Immune system boost. Essential nutrient intake	Infusion. Powder. Infusion
Monoplant recipe	*Paullinia pinnata* L.	Sapindaceae	Leaves. Roots	Supply of essential nutrients. Stimulation of appetite. Improvement of digestion. Fever. Strengthening of the immune system. Diarrhea. Cough. Intake of essential nutrients	Infusion. Decoction. Powder
Monoplant recipe	*Senna occidentalis* (L.) Link	Fabaceae	Leaves. Roots. Twigs. Flowers. Stems. Barks. Fruits. Seeds	Supply of essential nutrients. Fever. Cough. Convulsion. Immune system strengthening. Appetite stimulation. Digestion improvement. Diarrhea	Infusion. Trituration
Monoplant recipe	*Allium cepa* L.	Amaryllidaceae	Leaves	Supply of essential nutrients. Stimulation of appetite. Improved digestion. Strengthening of the immune system. Fever. Cough. Diarrhea	Infusion. Decoction. Powder. Trituration. Maceration
Monoplant recipe	*Arachis hypogaea* L.	Fabaceae	Leaves. Barks. Roots	Supply of essential nutrients. Fever. Diarrhea. Stimulation of appetite. Improved digestion. Strengthened immune system. Cough	Infusion. Trituration. Maceration
Monoplant recipe	*Croton gratissimus* Burch.	Euphorbiaceae	Leaves	Supply of essential nutrients. Stimulation of appetite. Convulsion. Fever. Cough. Improved digestion	Infusion
Monoplant recipe	*Erythrina senegalensis* DC.	Fabaceae	Leaves	Supply of essential nutrients. Stimulation of appetite. Fever. Convulsion. Improvement of digestion. Strengthening of the immune system	Infusion
Monoplant recipe	*Pavetta corymbosa* (DC.) F.N.Williams	Rubiaceae	Leaves	Supply of essential nutrients. Immune system boost. Digestion improvement. Fever. Appetite stimulation	Infusion
Monoplant recipe	*Psidium guajava* L.	Myrtaceae	Leaves. Barks	Diarrhea. Appetite stimulation. Essential nutrient intake. Fever. Cough	Infusion. Decoction
Monoplant recipe	*Pterocarpus erinaceus* Poir.	Fabaceae	Seeds. Barks	Supply of essential nutrients. Stimulation of appetite. Strengthening of the immune system. Fever. Improved digestion. Cough. Diarrhea. Others	Powder. Infusion. Maceration. Trituration. Decoction
Monoplant recipe	*Calotropis procera* (Aiton) Dryand.	Apocynaceae	Leaves. Barks. Roots	Supply of essential nutrients. Fever. Convulsion. Diarrhea	Infusion
Monoplant recipe	*Pavetta crassipes* K.Schum.	Rubiaceae	Barks	Fever. Appetite stimulation	Infusion
Monoplant recipe	*Musa × paradisiaca* L.	Musaceae	Leaves	Supply of essential nutrients. Appetite stimulation. Fever. Improved digestion	Infusion
Monoplant recipe	*Solanum macrocarpon* L.	Solanaceae	Leaves	Stimulation of appetite. Supply of essential nutrients. Fever. Strengthening of the immune system. Improvement of digestion	Infusion
Monoplant recipe	*Cymbopogon citratus* (DC.) Stapf	Poaceae	Leaves	Supply of essential nutrients. Stimulation of appetite. Fever. Improvement of digestion. Diarrhea. Strengthening of the immune system	Infusion. Decoction. Drink
Monoplant recipe	*Mesosphaerum suaveolens* (L.) Kuntze	Lamiaceae	Leaves	Supply of essential nutrients. Stimulation of appetite. Strengthening of the immune system. Convulsion. Diarrhea. Fever	Infusion
Monoplant recipe	*Spondias mombin* L.	Anacardiaceae	Leaves	Essential nutrients. Appetite stimulation. Fever. Cough. Convulsion. Supply of essential nutrients. Stimulation of appetite. Improved digestion. Strengthened immune system	Infusion
Monoplant recipe	*Aloe buettneri* A.Berger	Asphodelaceae	Leaves	Fever	Maceration
Monoplant recipe	*Annona senegalensis* Pers.	Annonaceae	Leaves. Fruits	Stimulation of appetite. Improved digestion. Supply of essential nutrients	Infusion
Monoplant recipe	*Crateva adansonii* DC.	Capparaceae	Leaves. Barks. Roots	Supply of essential nutrients. Immune system boost. Convulsion. Fever. Stimulation of appetite. Improved digestion. Cough	Infusion
Monoplant recipe	*Glycine max* (L.) Merr.	Fabaceae	Seeds	Supply of essential nutrients. Immune system support	Powder
Monoplant recipe	*Hibiscus sabdariffa* L.	Malvaceae	Leaves	Supply of essential nutrients. Convulsion. Improved digestion	Infusion
Monoplant recipe	*Landolphia dulcis* (Sabine ex G.Don) Pichon	Asphodelaceae	Fruits	Immune system boost. Digestion enhancement. Appetite stimulation. Essential nutrients. Fever. Diarrhea. Cough	Infusion. Trituration
Monoplant recipe	*Launaea taraxacifolia* (Willd.) Amin ex C.Jeffrey	Asteraceae	Leaves	Supply of essential nutrients. Stimulation of appetite. Fever. Diarrhea. Improved digestion	Trituration
Monoplant recipe	*Ocimum americanum* L.	Lamiaceae	Leaves. Roots	Strengthening of the immune system. Cough. Supply of essential nutrients. Fever	Infusion. Trituration
Monoplant recipe	*Oxytenanthera abyssinica* (A.Rich.) Munro	Poaceae	Leaves	Stimulation of appetite. Fever. Diarrhea. Supply of essential nutrients. Strengthening of the immune system	Infusion
Monoplant recipe	*Sterculia setigera* Delile	Malvaceae	Barks	Improve digestion. Provide essential nutrients. Stimulate appetite. Others	Infusion. Maceration. Trituration. Others
Monoplant recipe	*Vachellia seyal* (Delile) P.J.H. Hurter	Asphodelaceae	Leaves. Flowers. Fruits	Supply of essential nutrients. Stimulation of appetite. Fever. Convulsion. Immune system booster. Digestion enhancer. Cough. Diarrhea. Others	Infusion. Powder. Decoction. Trituration. Maceration
Monoplant recipe	*Ageratum conyzoides* L.	Asteraceae	Leaves	Stimulation of appetite. Improvement of digestion	Trituration
Monoplant recipe	*Azadirachta indica* A. Juss.	Meliaceae	Leaves	Fever. Others	Infusion
Monoplant recipe	*Rhodognaphalon brevicuspe* (Sprague) Roberty	Malvaceae	Leaves	Supply of essential nutrients. Stimulation of appetite. Improvement of digestion. Fever. Cough. Diarrhea	Infusion. Maceration
Monoplant recipe	*Sesamum radiatum* Thonn. ex Hornem.	Pedaliaceae	Leaves	Appetite stimulation	Infusion
Monoplant recipe	*Citrus maxima* (Burm.) Merr.	Rutaceae	Fruits. Others	Appetite stimulation	Infusion
Monoplant recipe	*Cleistopholis patens* (Benth.) Engl. & Diels	Annonaceae	Leaves	Fever. Diarrhea	Decoction
Monoplant recipe	*Cocos nucifera* L.	Arecaceae	Leaves	Stimulates appetite. Improves digestion. Strengthens the immune system. Diarrhea	Powder. Others
Monoplant recipe	*Combretum collinum* Fresen.	Combretaceae	Seeds	Appetite stimulation	Infusion
Monoplant recipe	*Ehretia cymosa* Thonn.	Boraginaceae	Leaves	Supply of essential nutrients. Strengthening of the immune system. Cough. Convulsion	Infusion
Monoplant recipe	*Elaeis guineensis* Jacq.	Arecaceae	Twigs. Fruits. Leaves	Supply of essential nutrients. Fever. Diarrhea. Cough. Stimulation of appetite. Improvement of digestion	Infusion. Decoction
Monoplant recipe	*Euphorbia hirta* L.	Euphorbiaceae	Leaves	Supply of essential nutrients. Immune system boost. Digestion improvement	Infusion
Monoplant recipe	*Ficus platyphylla* Delile	Moraceae	Leaves. Barks. Stems	Essential nutrients. Appetite stimulation. Digestion improvement. Fever. Immune system strengthening	Water. Honey. Oil. Infusion. Trituration. Powder
Monoplant recipe	*Heliotropium indicum* L.	Boraginaceae	Leaves	Supply of essential nutrients. Stimulation of appetite. Strengthening of the immune system. Cough. Diarrhea. Fever	Maceration. Trituration. Decoction. Powder. Infusion
Monoplant recipe	*Hibiscus cannabinus* L.	Malvaceae	Leaves. Barks	Supply of essential nutrients. Immune system boost. Diarrhea	Infusion
Monoplant recipe	*Hyphaene thebaica* (L.) Mart.	Arecaceae	Seeds	Fever	Infusion
Monoplant recipe	*Senna italica* Mill.	Fabaceae	Leaves	Supply of essential nutrients. Improvement of digestion. Stimulation of appetite. Strengthening of the immune system	Infusion
Monoplant recipe	*Irvingia gabonensis* (Aubry-Lecomte ex O'Rorke) Baill.	Fabaceae	Leaves. Barks	Supply of essential nutrients. Stimulation of appetite. Improved digestion	Infusion
Monoplant recipe	*Macrosphyra longistyla* (DC.) Hook.f. ex Hiern	Rubiaceae	Barks	Appetite stimulation. Fever	Decoction
Monoplant recipe	*Momordica balsamina* L.	Cucurbitaceae	Leaves. Stems. Flowers. Barks. Roots. Fruits. Branches. Seeds	Improved digestion. Strengthened immune system. Cough. Diarrhea. Fever. Supply of essential nutrients. Appetite stimulation. Convulsion	Infusion. Trituration. Maceration. Powder. Decoction
Monoplant recipe	*Newbouldia laevis *(P.Beauv.) Seem. ex Bureau	Bignoniaceae	Leaves	Supply of essential nutrients. Fever. Cough. Diarrhea	Infusion
Monoplant recipe	*Piliostigma thonningii* (Schumach.) Milne-Redh.	Fabaceae	Leaves	Diarrhea. Supply of essential nutrients. Immune system boost. Fever. Cough. Appetite stimulation	Infusion. Decoction
Monoplant recipe	*Cassia sieberiana* DC.	Fabaceae	Barks	Appetite stimulation. Diarrhea. Fever	Infusion. Trituration
Monoplant recipe	*Cola nitida* (Vent.) Schott & Endl	Malvaceae	Leaves. Stems. Flowers	Supply of essential nutrients. Fever. Diarrhea. Improved digestion	Infusion. Maceration
Monoplant recipe	*Blepharis linariifolia *Pers.	Acanthaceae	Aerial parts	Child care. Measles	Decoct and drink. Tisane. Decoct and wash
Monoplant recipe	*Hygrophila senegalensis *(Nees) T. Anders.	Acanthaceae	Seeds. Aerial parts	Jaundice. Extraction of foreign body from the eyes and ears	Suck in drops
Monoplant recipe	*Bauhinia rufescens *Lam.	Caesalpiniaceae	Aerial parts. Leaves. Roots. Barks	Smallpox. Diarrhea. Dysentery	Decoct and drink
Monoplant recipe	*Combretum glutinosum *Perr. ex DC.	Combretaceae	Leaves	Anemia. Stomachaches	Decoct and drink
Monoplant recipe	*Chrozophora brocchiana *Vis.	Euphorbiaceae	Whole plant. Roots. Leaves	Treatment for stomachaches in both children and adults	Decoct and drink. Suck in drops. Lapping powder
Monoplant recipe	*Phyllanthus pentandrus *Schumach. & Thonn.	Euphorbiaceae	Aerial parts. Leaves	Child weaning. Scorpion sting. Stomach pain. Indigestion	Decoct and drink
Monoplant recipe	*Bergia suffruticosa *(Delile) Fenzl	Elatinaceae	Aerial parts. Whole plant	Care for bones, wounds, stomach pains, common cold, and malaria	Decoct and drink. Decoct and wash
Monoplant recipe	*Gossypium herbaceum *L.	Malvaceae	Aerial parts. Flowers. Leafy stems	Skin diseases and infections. First aid remedy in the treatment of fever	Decoct and drink
Monoplant recipe	*Tephrosia lupinifolia *DC.	Papilionaceae	Aerial parts. Whole plant	Fortifier in the child. Stomachaches. Vomiting	Decoct and drink
Monoplant recipe	*Tephrosia linearis *(Willd.) Pers.	Papilionaceae	Aerial parts	Malaria	Decoct and drink
Monoplant recipe	*Indigofera leptoclada *Harms	Papilionaceae	Whole plant	Malaria. Breast abscess. Treatment of skin and mucous membrane diseases	Decoct and drink
Monoplant recipe	*Gardenia sokotensis *Hutch.	Rubiaceae	Whole plant. Twigs. Leaves. Roots. Barks (trunk)	Care of jaundice. Stomachaches. Malaria. Fever	Beverage. Decoct and drink. Powder
Monoplant recipe	*Ficus platyphylla *Delile	Moraceae	Branches	Supply of essential nutrients. Appetite stimulation. Cough. Diarrhea	Infusion. Maceration
Recipe with 2 plants	*Senna siamea *(Lam.) H.S.Irwin & Barneby	Fabaceae	Leaves	Stimulates appetite. Improves digestion. Fever	Infusion
	*Citrus × aurantiifolia *(Christm.) Swingle	Rutaceae	Fruits		
Recipe with 2 plants	*Spondias mombin *L.	Anacardiaceae	Leaves	Stimulation of appetite. Fever. Improved digestion. Diarrhea	Maceration
	*Cocos nucifera *L.	Arecaceae	Fruits		
Recipe with 2 plants	*Moringa oleifera *Lam.	Moringaceae	Leaves	Supply of essential nutrients. Improved digestion. Stimulation of appetite. Strengthening of the immune system	Decoction. Infusion
	*Glycine max *(L.) Merr.	Fabaceae	Grains		
Recipe with 2 plants	*Senna occidentalis *(L.) Link	Fabaceae	Leaves	Supply of essential nutrients. Improved digestion. Strengthened immune system. Fever. Convulsion. Cough	Infusion. Trituration
	*Khaya senegalensis *(Desr.) A. Juss.	Meliaceae	Barks		
Recipe with 2 plants	*Vitellaria paradoxa *C.F. Gaertn.	Sapotaceae	Leaves	Supply of essential nutrients. Stimulation of appetite. Fever. Strengthening of the immune system. Improvement of digestion	Infusion
	*Musa × paradisiaca* L.	Musaceae	Leaves		
Recipe with 2 plants	*Sterculia setigera *Delile	Malvaceae	Barks	Supply of essential nutrients. Stimulation of appetite. Improved digestion. Strengthened immune system. Fever	Infusion
	*Terminalia leiocarpa *(DC.) Baill.	Combretaceae	Leaves. Barks		
Recipe with 2 plants	*Sterculia setigera *Delile	Malvaceae	Barks	Essential nutrients. Appetite stimulation. Digestion enhancement. Immune system strengthening. Fever	Infusion
	*Pavetta crassipes *K.Schum.	Rubiaceae	Leaves. Barks		
Recipe with 2 plants	*Moringa oleifera *Lam.	Moringaceae	Leaves. Seeds	Supply of essential nutrients. Improved digestion. Strengthened immune system	Infusion. Trituration
	*Senna occidentalis *(L.) Link	Fabaceae	Leaves. Roots. Stems. Flowers. Seeds. Branches		
Recipe with 2 plants	*Moringa oleifera *Lam.	Moringaceae	Leaves	Supply of essential nutrients. Stimulation of appetite. Improved digestion. Strengthened immune system	Infusion
	*Elaeis guineensis* Jacq.	Arecaceae	Barks		
Recipe with 2 plants	*Ocimum gratissimum *L.	Lamiaceae	Leaves. Roots	Stimulation of appetite. Supply of essential nutrients. Improvement of digestion. Strengthening of the immune system. Fever	-
	*Vitellaria paradoxa *C.F. Gaertn.	Sapotaceae	Leaves		
Recipe with 2 plants	*Anacardium occidentale *L.	Anacardiaceae	Barks	Supply of essential nutrients. Stimulation of appetite. Improved digestion. Strengthened immune system. Fever. Cough	Infusion. Powder
	*Landolphia dulcis *(Sabine ex G.Don) Pichon	Asphodelaceae	Fruits		
Recipe with 2 plants	*Gymnanthemum amygdalinum *(Delile) Sch.Bip.	Asteraceae	Seeds	Essential nutrients. Fever. Diarrhea	Decoction
	*Senna occidentalis *(L.) Link	Fabaceae	Leaves		
Recipe with 2 plants	*Moringa oleifera *Lam.	Moringaceae	Leaves	Essential nutrients. Fever. Diarrhea	Decoction
	*Mangifera indica* L.	Anacardiaceae	Leaves		
Recipe with 2 plants	*Ocimum gratissimum *L.	Lamiaceae	Leaves. Barks	Essential nutrients. Diarrhea	Decoction. Powder. Maceration
	*Citrus × aurantiifolia *(Christm.) Swingle	Rutaceae	Fruits		
Recipe with 2 plants	*Pavetta crassipes *K.Schum.	Rubiaceae	Barks	Supply of essential nutrients. Diarrhea	Infusion
	*Carica papaya *L.	Caricaceae	Leaves		
Recipe with 2 plants	*Khaya senegalensis *(Desr.) A. Juss.	Meliaceae	Leaves. Barks	Supply of essential nutrients. Improved digestion. Fever. Diarrhea	Infusion
	*Senna occidentalis *(L.) Link	Fabaceae	Leaves		
Recipe with 2 plants	*Khaya senegalensis *(Desr.) A. Juss.	Meliaceae	Leaves. Barks	Supply of essential nutrients. Stimulates appetite. Improves digestion. Strengthens the immune system	Infusion
	*Pavetta crassipes *K.Schum.	Rubiaceae	Barks		
Recipe with 2 plants	*Carica papaya *L.	Caricaceae	Leaves	Supply of essential nutrients. Stimulation of appetite. Improved digestion. Strengthened immune system	Infusion
	*Moringa oleifera *Lam.	Moringaceae	Leaves		
Recipe with 2 plants	*Anonychium africanum *(Guill. & Perr.) C.E.Hughes & G.P.Lewis	Fabaceae	Leaves	Essential nutrients. Appetite stimulation	Infusion
	*Vitellaria paradoxa *C.F. Gaertn.	Sapotaceae	Leaves		
Recipe with 2 plants	*Piliostigma thonningii *(Schumach.) Milne-Redh.	Fabaceae	Leaves. Barks. Roots	Essential nutrients. Fever. Diarrhea	Infusion
	*Crateva adansonii *DC.	Capparaceae	Leaves. Barks		
Recipe with 2 plants	*Heliotropium indicum *L.	Boraginaceae	Leaves	Supply of essential nutrients. Stimulation of appetite. Improved digestion. Diarrhea	Infusion
	*Moringa oleifera *Lam.	Moringaceae	Leaves		
Recipe with 2 plants	*Moringa oleifera *Lam.	Moringaceae	Leaves	Diarrhea	Infusion
	*Cassytha filiformis *L.	Lauraceae	Leaves		
Recipe with 2 plants	*Momordica charantia *L.	Cucurbitaceae	Leaves	Fever. Convulsion. Cough. Diarrhea. Others	Infusion
	*Moringa oleifera *Lam.	Moringaceae	Roots		
Recipe with 2 plants	*Vitellaria paradoxa *C.F. Gaertn.	Sapotaceae	Leaves	Stimulation of appetite. Supply of essential nutrients. Strengthening of the immune system. Fever. Convulsion. Improvement of digestion	Infusion
	*Paullinia pinnata *L.	Sapindaceae	Leaves		
Recipe with 2 plants	*Glycine max *L.	Fabaceae	Seeds	Essential nutrients. Digestion enhancement. Immune system strengthening. Diarrhea	Powder
	*Combretum collinum *Fresen.	Combretaceae	Seeds		
Recipe with 2 plants	*Parinari curatellifolia *Planch. ex Benth.	Chrysobalanaceae	Leaves. Barks	Supply of essential nutrients. Fever. Diarrhea. Appetite stimulation	Decoction
	*Senna siamea *(Lam.) H.S.Irwin & Barneby	Fabaceae	Leaves		
Recipe with 2 plants	*Sterculia setigera *Delile	Malvaceae	Barks	Supply of essential nutrients. Stimulation of appetite. Improved digestion. Strengthened immune system	Infusion
	*Moringa oleifera *Lam.	Moringaceae	Leaves		
Recipe with 2 plants	*Pterocarpus erinaceus *Poir.	Fabaceae	Barks	Supply of essential nutrients. Improved digestion. Fever. Cough. Diarrhea. Strengthened immune system	Infusion
	*Carica papaya *L.	Caricaceae	Leaves		
Recipe with 2 plants	*Allium cepa *L.	Amaryllidaceae	Leaves	Supply of essential nutrients. Improves digestion. Stimulates appetite	Trituration
	*Moringa oleifera *Lam.	Moringaceae	Leaves		
Recipe with 2 plants	*Moringa oleifera *Lam.	Moringaceae	Leaves	Supply of essential nutrients. Improved digestion. Stimulation of appetite	Infusion
	*Elaeis guineensis* Jacq.	Arecaceae	Barks		
Recipe with 2 plants	*Senna siamea *(Lam.) H.S.Irwin & Barneby	Fabaceae	Leaves	Appetite stimulation. Digestion improvement. Fever	Infusion
	*Spondias mombin *L.	Anacardiaceae	Leaves		
Recipe with 2 plants	*Anonychium africanum *(Guill. & Perr.) C.E.Hughes & G.P.Lewis	Fabaceae	Leaves	Stimulation of appetite. Supply of essential nutrients. Improvement of digestion. Strengthening of the immune system	Infusion
	*Vitellaria paradoxa *C.F. Gaertn.	Sapotaceae	Leaves		
Recipe with 2 plants	*Senna occidentalis* (L.) Link	Fabaceae	Leaves	Supply of essential nutrients. Stimulates appetite. Improves digestion. Fever. Diarrhea	Infusion
	*Elaeis guineensis *Jacq.	Arecaceae	Barks		
Recipe with 2 plants	*Ficus platyphylla *Delile	Moraceae	Leaves. Stems. Barks	Stimulation of appetite. Supply of essential nutrients. Cough. Diarrhea. Fever	Infusion
	*Moringa oleifera *Lam.	Moringaceae	Leaves. Barks. Stems		
Recipe with 2 plants	*Moringa oleifera *Lam.	Moringaceae	Leaves	Supply of essential nutrients. Fever. Cough. Diarrhea. Stimulation of appetite. Improved digestion. Strengthened immune system	Decoction. Powder
	*Senna occidentalis *(L.) Link	Fabaceae	Leaves. Roots. Stems. Seeds		
Recipe with 2 plants	*Elaeis guineensis *Jacq.	Arecaceae	Barks	Supply of essential nutrients. Cough. Diarrhea. Fever. Stimulation of appetite. Improved digestion. Strengthened immune system	Decoction
	*Moringa oleifera *Lam.	Moringaceae	Leaves		
Recipe with 2 plants	*Elaeis guineensis *Jacq.	Arecaceae	Barks	Supply of essential nutrients. Stimulation of appetite. Improved digestion. Strengthened immune system. Fever	Infusion
	*Sterculia setigera* Delile	Malvaceae	Leaves		
Recipe with 2 plants	*Pavetta corymbosa *(DC.) F.N.Williams	Rubiaceae	Leaves	Supply of essential nutrients. Stimulation of appetite. Improved digestion. Strengthened immune system	Powder. Trituration
	*Senna italica *Mill.	Fabaceae	Leaves		
Recipe with 2 plants	*Monodora myristica *(Gaertn.) Dunal	Annonaceae	Fruits. Stems	Improved digestion. Diarrhea	Powder. Decoction. Infusion. Trituration. Maceration
	*Croton gratissimus *Burch.	Euphorbiaceae	Leaves		
Recipe with 2 plants	*Garcinia kola *Heckel	Clusiaceae	Barks. Roots. Flowers	Supply of essential nutrients. Improved digestion. Fever	Maceration. Trituration. Powder. Infusion. Decoction
	*Newbouldia laevis *(P.Beauv.) Seem. ex Bureau	Bignoniaceae	Leaves		
Recipe with 2 plants	*Cola nitida *(Vent.) Schott & Endl	Malvaceae	Flowers. Stems. Roots	Supply of essential nutrients. Digestion improved. Immune system strengthened. Fever	Infusion. Powder. Trituration. Maceration. Decoction
	*Mentha × piperita *L.	Lamiaceae	Leaves		
Recipe with 2 plants	*Ocimum americanum *L.	Lamiaceae	Barks	Immune system boost. Convulsion	Maceration. Trituration. Decoction
	*Xylopia aethiopica *(Dunal) A.Rich.	Annonaceae	Stems. Fruits		
Recipe with 2 plants	*Zingiber officinale *Roscoe	Zingiberaceae	Leaves	Appetite stimulant. Immune system booster	Maceration. Trituration. Decoction. Powder. Infusion
	*Xylopia aethiopica* (Dunal) A.Rich.	Annonaceae	Roots. Stems		
Recipe with 2 plants	*Kalanchoe pinnata* (Lam.) Pers.	Crassulaceae	Bark	Strengthens the immune system. Cough	Decoction. Trituration. Maceration
	*Xylopia aethiopica* (Dunal) A.Rich.	Annonaceae	Stems. Flowers		
Recipe with 2 plants	*Calotropis procera* (Aiton) Dryand.	Apocynaceae	Barks. Stems	Essential nutrients. Diarrhea. Fever. Improved digestion	Maceration. Infusion
	*Xylopia aethiopica* (Dunal) A.Rich.	Annonaceae	Flowers. Fruits		
Recipe with 2 plants	*Moringa oleifera *Lam.	Moringaceae	Leaves	Diarrhea. Supply of essential nutrients. Stimulation of appetite. Strengthening of the immune system	Infusion. Maceration
	*Crateva adansonii *DC.	Capparaceae	Barks		
Recipe with 2 plants	*Croton gratissimus* Burch.	Euphorbiaceae	Leaves	Essential nutrients. Appetite stimulation. Cough. Diarrhea	Infusion. Maceration
	*Calotropis procera *(Aiton) Dryand.	Apocynaceae	Leaves		
Recipe with 2 plants	*Khaya senegalensis *(Desr.) A. Juss.	Meliaceae	Leaves	Supply of essential nutrients. Appetite stimulation. Cough. Diarrhea	Infusion. Maceration
	*Senna siamea *(Lam.) H.S.Irwin & Barneby	Fabaceae	Barks		
Recipe with 2 plants	*Mangifera indica *L.	Anacardiaceae	Leaves	Essential nutrients. Diarrhea. Fever. Improved digestion	Maceration. Infusion
	*Hibiscus cannabinus *L.	Malvaceae	Leaves		
Recipe with 2 plants	*Allium cepa *L.	Amaryllidaceae	Leaves	Essential nutrients. Appetite stimulation. Fever. Convulsion. Cough. Diarrhea	Decoction. Infusion. Powder. Trituration
	*Momordica balsamina *L.	Cucurbitaceae	Leaves. Flowers. Fruits		
Recipe with 2 plants	*Moringa oleifera *Lam.	Moringaceae	Leaves	Supply of essential nutrients. Stimulation of appetite. Improved digestion. Strengthened immune system. Fever	Infusion
	*Pterocarpus erinaceus *Poir.	Fabaceae	Barks		
Recipe with 2 plants	*Ximenia americana *L.	Olacaceae	Barks	Supply of essential nutrients. Stimulation of appetite. Fever. Diarrhea	Infusion
	*Moringa oleifera *Lam.	Moringaceae	Leaves		
Recipe with 2 plants	*Senna occidentalis *(L.) Link	Fabaceae	Leaves. Roots. Stems. Seeds	Supply of essential nutrients. Stimulation of appetite. Fever. Diarrhea	Infusion
	*Pterocarpus erinaceus *Poir.	Fabaceae	Barks		
Recipe with 2 plants	*Moringa oleifera *Lam.	Moringaceae	Leaves	Supply of essential nutrients. Stimulation of appetite. Fever. Diarrhea	Infusion
	*Allium cepa *L.	Amaryllidaceae	Leaves		
Recipe with 2 plants	*Momordica charantia *L.	Cucurbitaceae	Barks. Roots. Fruits. Branches	Essential nutrients	Infusion. Maceration. Decoction. Powder. Trituration
	*Nauclea latifolia *Sm.	Rubiaceae	Flowers. Stems. Leaves		
Recipe with 2 plants	*Adansonia digitata *L.	Malvaceae	Leaves	Supply of essential nutrients. Improved digestion. Strengthened immune system. Fever	Infusion. Powder. Trituration. Maceration. Decoction
	*Moringa oleifera *Lam.	Moringaceae	Leaves		
Recipe with 2 plants	*Moringa oleifera *Lam.	Moringaceae	Leaves	Supply of essential nutrients. Stimulation of appetite. Improvement of digestion	Infusion
	*Elaeis guineensis *Jacq.	Arecaceae	Barks		
Recipe with 3 plants	*Senna occidentalis *(L.) Link	Fabaceae	Leaves	Stimulation of appetite. Supply of essential nutrients. Improvement of digestion. Strengthening of the immune system	Infusion
	*Moringa oleifera *Lam.	Moringaceae	Leaves		
	*Elaeis guineensis *Jacq.	Arecaceae	Leaves. Barks		
Recipe with 3 plants	*Moringa oleifera *Lam.	Moringaceae	Leaves	Supply of essential nutrients. Stimulation of appetite. Improved digestion. Strengthened immune system	Infusion
	*Elaeis guineensis* Jacq.	Arecaceae	Barks		
	*Sterculia setigera* Delile	Malvaceae	Barks		
Recipe with 3 plants	*Carica papaya *L.	Caricaceae	Leaves	Supply of essential nutrients. Stimulation of appetite. Improved digestion. Strengthened immune system. Diarrhea	Infusion
	*Senna occidentalis *(L.) Link	Fabaceae	Leaves		
	*Pavetta crassipes *K.Schum.	Rubiaceae	Barks		
Recipe with 3 plants	*Manihot esculenta* Crantz	Euphorbiaceae	Leaves	Supply of essential nutrients. Appetite stimulation	Infusion
	*Carica papaya *L.	Caricaceae	Leaves		
	*Allium cepa *L.	Amaryllidaceae	Fruits		
Recipe with 3 plants	*Senna siamea *(Lam.) H.S.Irwin & Barneby	Fabaceae	Leaves	Supply of essential nutrients. Stimulation of appetite. Improved digestion. Fever	Infusion
	*Elaeis guineensis *Jacq.	Arecaceae	Barks		
	*Moringa oleifera *Lam.	Moringaceae	Leaves		
Recipe with 3 plants	*Senna occidentalis *(L.) Link	Fabaceae	Leaves	Supply of essential nutrients. Stimulation of appetite. Improvement of digestion. Strengthening of the immune system. Fever	Infusion
	*Sterculia setigera *Delile	Malvaceae	Barks		
	*Pavetta crassipes *K.Schum.	Rubiaceae	Leaves. Barks		
Recipe with 3 plants	*Vitellaria paradoxa* C.F. Gaertn.	Sapotaceae	Leaves	Convulsion. Immune system boost. Diarrhea	Decoction
	*Paullinia pinnata *L.	Sapindaceae	Leaves		
Recipe with 3 plants	*Elaeis guineensis* Jacq.	Arecaceae	Barks	Essential nutrients. Stimulate appetite. Improve digestion. Strengthen the immune system. Fever	Infusion
	*Sterculia setigera* Delile	Malvaceae	Barks		
	*Pavetta crassipes* K.Schum.	Rubiaceae	Leaves. Barks		
Recipe with 3 plants	*Moringa oleifera *Lam.	Moringaceae	Leaves	Supply of essential nutrients. Stimulates appetite. Improves digestion. Strengthens the immune system. Fever. Diarrhea	Infusion
	*Elaeis guineensis* Jacq.	Arecaceae	Barks		
	*Pavetta crassipes *K.Schum.	Rubiaceae	Barks		
Recipe with 3 plants	*Moringa oleifera *Lam.	Moringaceae	Leaves	Supply of essential nutrients. Stimulation of appetite. Improved digestion. Fever	Infusion
	*Sterculia setigera *Delile	Malvaceae	Barks		
	*Senna occidentalis *(L.) Link	Fabaceae	Leaves		
Recipe with 3 plants	*Moringa oleifera *Lam.	Moringaceae	Leaves	Supply of essential nutrients. Stimulation of appetite. Improved digestion. Strengthened immune system. Fever	Infusion
	*Carica papaya *L.	Caricaceae	Leaves		
	*Elaeis guineensis *Jacq.	Arecaceae	Barks		
Recipe with 3 plants	*Setaria sphacelata *(Schumach.) Stapf & C.E.Hubb. ex Moss	Poaceae	Leaves. Roots	Supply of essential nutrients. Stimulates appetite. Improves digestion. Fever. Strengthens the immune system	Infusion
	*Ocimum gratissimum *L.	Lamiaceae	Leaves		
	*Paullinia pinnata *L.	Sapindaceae	Leaves		
Recipe with 3 plants	*Sterculia setigera *Delile	Malvaceae	Barks	Stimulation of appetite. Improved digestion. Fever. Diarrhea	Infusion
	*Elaeis guineensis *Jacq.	Arecaceae	Barks		
	*Moringa oleifera *Lam.	Moringaceae	Barks. Leaves		
Recipe with 3 plants	*Carica papaya *L.	Caricaceae	Leaves	Supply of essential nutrients. Stimulation of appetite. Fever. Diarrhea	Infusion
	*Senna occidentalis *(L.) Link	Fabaceae	Leaves		
	*Moringa oleifera *Lam.	Moringaceae	Leaves		
Recipe with 3 plants	*Terminalia leiocarpa *(DC.) Baill.	Combretaceae	Leaves. Barks	Supply of essential nutrients. Cough. Diarrhea. Fever	Decoction
	*Moringa oleifera *Lam.	Moringaceae	Leaves		
	*Sterculia setigera *Delile	Malvaceae	-		
Recipe with 3 plants	*Uvaria chamae *P.Beauv.	Annonaceae	Barks	Stimulation of appetite. Strengthening of the immune system	Trituration. Maceration
	*Xylopia aethiopica *(Dunal) A.Rich.	Annonaceae	Flowers. Stems		
	*Croton gratissimus* Burch.	Euphorbiaceae	Leaves. Roots		
Recipe with 3 plants	*Jatropha curcas *L.	Euphorbiaceae	Leaves	Diarrhea. Stimulation of appetite. Improved digestion. Supply of essential nutrients	Infusion
	*Carica papaya *L.	Caricaceae	Roots		
	*Arachis hypogaea *L.	Fabaceae	Barks		
Recipe with 3 plants	*Arachis hypogaea *L.	Fabaceae	Leaves	Supply of essential nutrients. Stimulation of appetite. Diarrhea. Improved digestion	Infusion. Maceration
	*Hibiscus cannabinus *L.	Malvaceae	Leaves		
	*Crateva adansonii *DC.	Capparaceae	Leaves		
Recipe with 3 plants	*Moringa oleifera *Lam.	Moringaceae	Leaves	Supply of essential nutrients. Convulsion. Diarrhea	Decoction. Infusion. Trituration. Maceration
	*Anonychium africanum *(Guill. & Perr.) C.E.Hughes & G.P.Lewis	Fabaceae	Leaves		
	*Mangifera indica *L.	Anacardiaceae	Leaves		
Recipe with 3 plants	*Khaya senegalensis *(Desr.) A. Juss.	Meliaceae	Leaves	Essential nutrients. Fever. Appetite stimulation. Digestion improvement	Infusion
	*Phyllanthus amarus *Schumach. & Thonn.	Phyllanthaceae	Barks		
	*Carica papaya *L.	Caricaceae	Roots		
Recipe with 3 plants	*Carica papaya *L.	Caricaceae	Leaves	Supply of essential nutrients. Stimulation of appetite. Improved digestion. Diarrhea. Strengthening of the immune system	Infusion
	*Khaya senegalensis *(Desr.) A. Juss.	Meliaceae	Barks		
	*Hibiscus cannabinus *L.	Malvaceae	-		
Recipe with 3 plants	*Crateva adansonii *DC.	Capparaceae	Leaves	Supply of essential nutrients. Stimulation of appetite. Improved digestion. Fever. Convulsion. Cough	Infusion
	*Ocimum gratissimum *L.	Lamiaceae	Barks		
	*Senna siamea *(Lam.) H.S.Irwin & Barneby	Fabaceae	Roots		
Recipe with 3 plants	*Adansonia digitata *L.	Malvaceae	Leaves	Supply of essential nutrients. Stimulation of appetite. Improved digestion. Fever. Cough. Diarrhea	Infusion. Decoction. Trituration
	*Ocimum gratissimum *L.	Lamiaceae	Leaves. Roots		
	*Moringa oleifera *Lam.	Moringaceae	Roots		
Recipe with 3 plants	*Croton gratissimus *Burch.	Euphorbiaceae	Leaves	Supply of essential nutrients. Cough. Diarrhea. Improved digestion	Decoction. Maceration
	*Phyllanthus amarus *Schumach. & Thonn.	Phyllanthaceae	-		
	*Crateva adansonii *DC.	Capparaceae	-		
Recipe with 3 plants	*Moringa oleifera *Lam.	Moringaceae	Leaves	Improved digestion	Infusion
	*Senna occidentalis *(L.) Link	Fabaceae	Leaves		
	*Elaeis guineensis *Jacq.	Arecaceae	Barks		
Recipe with 3 plants	*Moringa oleifera *Lam.	Moringaceae	Leaves	Essential nutrients. Appetite stimulation. Diarrhea	Infusion. Decoction. Trituration. Maceration
	*Crateva adansonii *DC.	Capparaceae	Leaves		
	*Psidium guajava *L.	Myrtaceae	Leaves		
Recipe with 3 plants	*Ocimum gratissimum *L.	Lamiaceae	Leaves	Essential nutrients. Appetite stimulating. Diarrhea	Infusion. Decoction. Trituration. Maceration
	*Crateva adansonii *DC.	Capparaceae	Roots		
	*Hibiscus cannabinus *L.	Malvaceae	-		
Recipe with 3 plants	*Senna occidentalis *(L.) Link	Fabaceae	Leaves	Supply of essential nutrients. Appetite stimulation. Diarrhea	Infusion. Decoction. Trituration. Maceration
	*Sterculia setigera *Delile	Malvaceae	Barks		
	*Moringa oleifera *Lam.	Moringaceae	Barks		
Recipe with 3 plants	*Senna occidentalis *(L.) Link	Fabaceae	Leaves	Supply of essential nutrients. Improved digestion. Fever. Diarrhea	Infusion
	*Sterculia setigera *Delile	Malvaceae	Barks		
	*Moringa oleifera *Lam.	Moringaceae	Barks		
Recipe with 3 plants	*Moringa oleifera *Lam.	Moringaceae	Leaves	Improves digestion	Infusion
	*Elaeis guineensis* Jacq.	Arecaceae	Barks		
	*Sterculia setigera* Delile	Malvaceae	Leaves		
Recipe with 3 plants	*Elaeis guineensis *Jacq.	Arecaceae	Barks	Supply of essential nutrients. Stimulation of appetite. Improved digestion. Strengthened immune system. Fever	Infusion
	*Pavetta crassipes* K.Schum.	Rubiaceae	Barks		
	*Moringa oleifera *Lam.	Moringaceae	Seeds		
Recipe with 4 plants	*Senna occidentalis *(L.) Link	Fabaceae	Leaves	Supply of essential nutrients. Stimulation of appetite. Improved digestion. Strengthened immune system. Diarrhea	Infusion
	*Moringa oleifera *Lam.	Moringaceae	Leaves		
	*Sterculia setigera *Delile	Malvaceae	Barks		
	*Elaeis guineensis *Jacq.	Arecaceae	Barks		
Recipe with 4 plants	*Moringa oleifera *Lam.	Moringaceae	Leaves	Supply of essential nutrients. Stimulation of appetite. Improved digestion. Fever	Infusion
	*Elaeis guineensis* Jacq.	Arecaceae	Barks		
	*Terminalia leiocarpa *(DC.) Baill.	Combretaceae	Leaves. Barks		
	*Senna occidentalis *(L.) Link	Fabaceae	Leaves		
Recipe with 4 plants	*Senna siamea *(Lam.) H.S.Irwin & Barneby	Fabaceae	Leaves. Roots	Appetite stimulation	Infusion
	*Jatropha curcas *L.	Euphorbiaceae	Leaves		
	*Cocos nucifera *L.	Arecaceae	Barks		
	*Cymbopogon citratus *(DC.) Stapf	Poaceae	Leaves		
Recipe with 4 plants	*Calotropis procera *(Aiton) W.T. Aiton	-	Leaves	Supply of essential nutrients. Stimulation of appetite. Diarrhea. Strengthening of the immune system	Infusion. Maceration
	*Anonychium africanum *(Guill. & Perr.) C.E.Hughes & G.P.Lewis	Fabaceae	Leaves. Roots. Barks		
	*Hibiscus cannabinus *L.	Malvaceae	Roots		
	*Newbouldia laevis *(P.Beauv.) Seem. ex Bureau	Bignoniaceae	Barks		
Recipe with 4 plants	*Senna siamea *(Lam.) H.S.Irwin & Barneby	Fabaceae	Leaves	Supply of essential nutrients. Stimulation of appetite. Improved digestion. Convulsion. Diarrhea	Infusion
	*Chromolaena odorata *(L.) R.M.King & H.Rob	Rubiaceae	Barks		
	*Hibiscus cannabinus *L.	Malvaceae	-		
	*Crateva adansonii *DC.	Capparaceae	-		
Recipe with 4 plants	*Hibiscus cannabinus *L.	Malvaceae	Roots	Supply of essential nutrients. Stimulation of appetite. Improved digestion. Fever. Cough. Diarrhea	Infusion. Decoction. Trituration
	*Senna siamea *(Lam.) H.S.Irwin & Barneby	Fabaceae	Barks		
	*Moringa oleifera *Lam.	Moringaceae	Leaves		
	*Khaya senegalensis *(Desr.) A. Juss.	Meliaceae	Leaves		
Recipe with 5 plants	*Pavetta crassipes *K.Schum.	Rubiaceae	Barks	Supply of essential nutrients. Stimulation of appetite. Improved digestion. Strengthened immune system. Fever. Diarrhea	Infusion
	*Elaeis guineensis *Jacq.	Arecaceae	Barks		
	*Moringa oleifera *Lam.	Moringaceae	Leaves		
	*Sterculia setigera *Delile	Malvaceae	Barks		
	*Khaya senegalensis *(Desr.) A. Juss.	Meliaceae	Barks		
Recipe with 5 plants	*Pavetta crassipes *K.Schum.	Rubiaceae	Leaves. Barks	Supply of essential nutrients. Stimulation of appetite. Improvement of digestion. Strengthening of the immune system. Fever. Diarrhea	Infusion
	*Moringa oleifera *Lam.	Moringaceae	Leaves		
	*Senna occidentalis *(L.) Link	Fabaceae	Leaves		
	*Elaeis guineensis *Jacq.	Arecaceae	Barks		
	*Sterculia setigera* Delile	Malvaceae	Barks		
Recipe with 5 plants	*Senna siamea *(Lam.) H.S.Irwin & Barneby	Fabaceae	Barks. Roots	Supply of essential nutrients. Stimulation of appetite. Improvement of digestion	Powder. Infusion. Maceration. Trituration. Decoction
	*Hibiscus acetosella *Welw. ex Hiern	Malvaceae	Flowers. Fruits		
	*Newbouldia laevis *(P.Beauv.) Seem. ex Bureau	Bignoniaceae	Leaves		
	*Momordica charantia *L.	Cucurbitaceae	Leaves		
	*Carica papaya *L.	Caricaceae	Leaves		
